# Adverse effects of non-hormonal pharmacological interventions in breast cancer survivors, suffering from hot flashes: A systematic review and meta-analysis

**DOI:** 10.1007/s10549-016-4002-x

**Published:** 2016-10-05

**Authors:** Jill Brook Hervik, Trine Stub

**Affiliations:** 1 Department of Anesthesia, Pain Clinic, Vestfold Hospital Trust, 3116 Tonsberg, Norway; 2 The National Research Center in Complementary and Alternative Medicine (NAFKAM) Department of Community Medicine, Faculty of Health Science, UiT The Arctic University of Norway, 9037 Tromso, Norway

**Keywords:** Adverse effects, Non-hormonal drugs, Breast cancer, Hot flashes

## Abstract

**Purpose:**

To access frequency and severity of adverse effects (AE) of non-hormonal drugs (NHD) for hot flashes in breast cancer survivors compared to controls and analyze adverse-effect risk by reviewing published randomized trials.

**Methods:**

Cochrane Central Register for Controlled Trials, Embase, Medline, PsycINFO and PubMed databases were searched. Trials were included where participants were survivors of breast cancer suffering from hot flashes, treatment included self-administered venlafaxine, gabapentin or clonidine, and AE were reported. AE frequency and severity were graded. A meta-analysis of ten trials with sub-group analyses was conducted.

**Results:**

Forty-nine studies were identified, and 12 were included. A total of 1467 participants experienced 772 adverse effects, 81 % (*n* = 627) in the treatment group and 19 % (*n* = 145) in the control group. Sixty-seven percent of AE was graded as mild and 33 % as moderate. The frequency of AE for NHD was overall significant compared to placebo. Sub-group analysis indicated that AE frequency and severity increased at higher doses of venlafaxine and gabapentin compared to placebo.

**Conclusion:**

The odds for experiencing AE was significantly higher in patients randomized to high-dose NHD than those randomized to controls, including placebo, low-dose medication and acupuncture. These therapies should be considered as a potential treatment alternative.

## Introduction

Breast cancer is the second most common cancer in the world and the most frequent cancer among women. 1.67 million new cases were diagnosed in 2012 [1].

Treatment of breast cancer includes surgery, chemotherapy, radiation and endocrine therapy. Fifty percent of women diagnosed with breast cancer have a tumour that is oestrogen receptor positive, and consequently, they are offered hormone-suppression treatment lasting for at least five years [2]. Tamoxifen is an oestrogen receptor modulator which blocks the effect of oestrogen in breast tissue. It is indicated for use in premenopausal women and, as an initial treatment, in post-menopausal women. Aromatase inhibitors are recommended only for post-menopausal women, in whom the main source of oestrogen comes from the conversion of testosterone to estradiol, facilitated by the aromatase enzyme.

A common adverse effect of oestrogen-antagonist therapy is hot flashes. Up to 80 % of women medicated with tamoxifen suffer from hot flashes, 30 % of which rate them as severe [3, 4]. Severe hot flash problems can result in women stopping potentially lifesaving oestrogen-antagonist treatments; up to 25 % of women with breast cancer do not adhere to adjuvant oestrogen-antagonist therapy [5]. Consequently, better management of adverse effects including hot flashes is important for increasing compliance and achieving optimal results.

Self-administered treatments for hot flash problems such as drugs, creams or patches are the easiest and most practical therapy for most women. The most effective treatment is oestrogen therapy, but it is not recommended in women with breast cancer, and no safe conclusions regarding the use of progesterone are available [6]. Sixty percent of breast cancer tumours are oestrogen and/or progesterone receptor positive and therefore responsive to hormonal influence [7]. Contraindications surrounding hormonal therapies for the treatment of menopausal symptoms in breast cancer survivors have provoked increased use of non-hormonal drugs. Non-hormonal treatment includes therapies that do not affect oestrogen or progesterone production or action [8]. Self-administered therapies including anti-hypertensive medications, selective serotonin reuptake inhibitors (SSRI), selective norepinephrine reuptake inhibitors (SNRI), and anticonvulsant medicines have been studied for hot flash symptoms and increasingly used during the last decade. The most commonly used drugs in this category include venlafaxine, a selective serotonin reuptake inhibitor; the anticonvulsant gabapentin; and clonidine a centrally acting antiadrenergic agent, commonly used to control hypertension.

Randomized controlled trials (RCT) of drugs in these categories are limited; however, two systematic reviews have reported on the efficacy of these three drugs as a treatment for hot flashes in both breast cancer survivors and healthy menopausal women [8, 9]. Paroxetine and Fluoxetine, both being SSRIs, have also shown efficacy in the reduction of hot flashes [10–13]; however, these drugs interfere with the metabolization of tamoxifen to endoxifen [10, 14] and are therefore contraindicated in women using tamoxifen.

Various complementary and alternative therapies have been studied as a treatment for HF in breast cancer patients. Vitamin E has not demonstrated efficacy [8], while phytoestrogens possibly involve oestrogenic influence and are therefore not recommended for women with breast cancer [15]. Controversy around the safety of Cimicifuga Racemosa (Black Cohosh) as a treatment for menopausal symptoms exists because of its purported oestrogenic activity. A systematic review of 26 articles concluded that current evidence does not support an association between black cohosh and increased risk of breast cancer, and those conflicting but promising results for the reduction of HF in breast cancer patients warrant the need for further research [16]. Cognitive behavioural therapy trials [17, 18] and relaxation [19] have shown modest, short-term effect. Two trials investigating the effect of homoeopathy versus placebo [8], neither were RCT, found a statistically significant improvement in HF frequency for homoeopathy over placebo. Acupuncture was as effective as venlafaxine in a trial comparing these two interventions. However, 18 incidences of adverse effects were recorded in the venlafaxine group, whereas the acupuncture group experienced no adverse effects [20]. A systematic review of acupuncture to control hot flashes, which included 8 breast cancer studies (*n* = 474), concluded that the current level of evidence is insufficient to support the treatment of hot flashes [21].

## The importance of this review

The efficacy and adverse-effect profiles of hot flash treatment vary in non-hormonal pharmacological interventions. Comparing studies of interventions in this category may provide an indication as to whether treatment effect outweighs adverse effects in breast cancer survivors. Potential information regarding the tolerability of each drug has direct clinical implications, affecting decision making and compliance.

## Aims

The aims of this review are to

1. systematically investigate how adverse effects of the three most commonly used non-hormonal drugs, to treat hot flashes in breast cancer patients, are reported in randomized controlled trials;
2. classify adverse effects and drug-related aggravations according to the Common Terminology Criteria for Adverse Effects (CTCAE) [22] and
3. perform a meta-analysis to evaluate the risk of adverse effects for patients pharmacologically managing their hot flashes with non-hormonal self-administered therapy, compared to different controls.

## Terminology

If a substance is capable of producing a therapeutic effect, it can also produce harmful or unwanted effects. Terms used to describe such unwanted effects include side effect, adverse effect, adverse event, adverse reaction and toxic effect [23]. The term *adverse effect* used in this paper is defined by *The European Medicines Agency* [24] as *any untoward medical occurrence in a patient or clinical trial subject administered a medical product.* This term encompasses all unwanted effects, without making assumptions about their mechanism [25].

## Methods

### Search methods for identification of studies

The focus question was: *Are the most commonly used non*-*hormonal drugs for hot flashes in breast cancer patients associated with adverse effects?* The four elements from PICO were used when searching for relevant articles:

1. Population: Patients with breast cancer, suffering from hot flashes.
2. Intervention: Non-hormonal self-administered pharmacological therapies, including venlafaxine, gabapentin and clonidine.
3. Comparison: Placebo, other non-hormonal drugs, conventional medical therapies, CAM, waiting list and usual care.
4. Outcome: Adverse effects, adverse events, adverse reactions, tolerability, side effects and toxicity.

The following electronic databases were searched with no language, publication, or time restrictions: Cochrane Central Register for Controlled Trials (Central) in the Cochrane library, Embase, Medline, PsycINFO and PubMed.

Titles and abstracts were identified through the search strategy. If no abstract was available, the full text paper was obtained for inspection. Both authors did the searches, read the articles and extracted the data (search strings are attached in the appendix). Grey literature was searched in order to find possibly missed articles through electronic searches. References of all retrieved articles and systematic reviews were searched [8, 9, 26–28]. Depending on the database, various combinations of MESH terms and keywords were used. MESH terms included* breast neoplasms, breast cancer, hot flashes, clonidine, adverse effect, adverse drug reaction reporting systems*. The following keywords were applied: *breast cancer, hot flash, hot flush, vasomotor symptom, clonidine, venlafaxine, gabapentin, adverse effect, adverse event and side*-*effect*.

Inclusion comprised randomized controlled trials that reported adverse effects of treatment. Both parallel group design and cross-over studies were included. Data from cross-over studies were included from both treatment periods, since all cross-over studies specified that there was no cross-over effect.

Data were extracted to give information on the total number of adverse effects and number of patients experiencing the adverse effects. Severity of adverse effects was assessed using the CTCAE grading system and was entirely dependent on the information provided in the articles. The system grades adverse effects from 1 to 5, where 1 indicates mild symptoms, 2 moderate symptoms, 3 severe symptoms, 4 life threatening and 5 fatal symptoms. When summarizing the data, the total number of adverse effects was counted, regardless of the number of participants experiencing them. Both authors categorized and graded the data. Lack of consensus was settled by discussion.

A methodological assessment including risk of bias was made by both authors using criteria from the Cochrane Handbook of Systematic Reviews and Interventions [29]. The trials were rated as follows:

A grading of “A” indicates a RCT of high quality with low risk of bias with adequate measures to conceal allocation, detailed randomization description and implementation of the intention to treat principle.

Grade “B” was used when method of allocation concealment was not described, or was unclear, creating a moderate risk of bias.

A grade “C” was used when the method of allocation was not concealed; such trials were excluded because of high risk of bias.

Extracted data included number of patients randomized to each group, number of dropouts, use of power calculation, whether the intention to treat principle was followed, intervention (including dose), duration of intervention, main findings and funding. The authors of retrieved articles were contacted when in doubt of or there is a lack of information in the publications (Table 1).

**Table 1:** Assessment of the methodological quality of the randomized controlled trials

Study ID	Indication	Participants		Dropout		PC/ITT analyses	Methodological assessment	Intervention	Duration of treatment	Main findings	Funding
		*Treatment*	*Control*	*Treatment*	*Control*		*Cochrane Handbook*	*Treatment vs Control*			
Buijs [36]	Venlafaxine versus clonidine for hot flashes in women with breast cancer	Venlafaxine/clonidine *n* = 30	Clonidine/venlafaxine *n* = 30	Venlafaxine period *n* = 15	Clonidine period *n* = 5	Yes/Yes	A-clear	Venlafaxine (75 mg × 1) Clonidine (0.05 mg x 2) Cross-over design. Randomized Double blind	18 weeks - 2 × 8 weeks with 2 week wash out period	Venlafaxine and clonidine were equally effective in hot flash reduction. Main reasons for discontinuation were adverse effects, which were worse with venlafaxine	NR
Biglia [39]	Gabapentin versus vitamin E for hot flashes and sleep quality in breast cancer patients	Gabapentin *n* = 60	Vitamin E *n* = 55	29	25	Yes/Yes	B-unclear. No allocation concealment or blinding	Gabapentin 900 mg/day vs Vitamin E 800 IU/day. Parallel group design, randomized but not blinded	12 weeks + 12 week obs.	Gabapentin (900 mg) significantly reduced hot flash frequency and score, a non-significant reduction was seen in the vitamin E group	Not funded
Boekhout [31]	Management of hot flashes in breast cancer patients with venlafaxine and clonidine	Venlafaxine *n* = 41 Clonidine *n* = 41	Placebo *n* = 20	Venlafaxine * n* = 6 Clonidine * n* = 13	3	Yes/Yes	A-clear	Venlafaxine 75 mg/day vs Clonidine 0.1 mg/day vs placebo. Parallel group design. Stratified randomization. Double blind	12 weeks	Venlafaxine and clonidine are effective treatments for hot flashes in breast cancer patients. A more immediate reduction was seen with venlafaxine, however hot flash scores were lower with clonidine at week 12	NR
Carpenter [32]	Dose-related efficacy of venlafaxine in the treatment of hot flashes in breast cancer patients	Venlafaxine 37.5/placebo *n* = 64.	Venlafaxine 75 mg/placebo * n* = 20	21	3	Yes/Yes	A-clear	Venlafaxine low and high-dose groups randomized to 2 sequences each, 6 weeks treatment then 6 weeks placebo or visa versa. Cross-over design, randomized, double blind	12 weeks	Venlafaxine resulted in modest decreases in hot flashes, only hot flash interference improved at the higher dose. Although adverse effects were mild most women discontinued venlafaxine long-term possibly due to treatments not outweighing benefits	National Institute of Nursing Research. USA
Goldberg [38]	Transdermal clonidine for tamoxifen-induced hot flashes	Clonidine/placebo *n* = 55	Placebo/clonidine *n* = 55	13	8	No/Yes	A-clear	4 weeks of transdermal clonidine (equivalent to 0.1 mg oral dose), the 4 weeks placebo or visa versa. Cross-over, randomized, double-blind design	8 weeks	Clonidine significantly reduced hot flash frequency and severity, 4 different adverse effects were recorded	Public Health Service Grants. USA
Liobl [42]	Venlafaxine versus clonidine for hot flashes in breast cancer patients	Venlafaxine *n* = 40	Clonidine *n* = 40	9	7	Yes/Yes	A-clear	Venlafaxine (37.5 mg x 2) Clonidine (0.075 mg x 2) Double-blind, randomized, cross-over design, only 39 patients were crossed over at week 4.	Part A = 4 weeks Part B (cross-over) = 8 weeks. No wash out period	Venlafaxine is significantly more effective in reducing the frequency of hot flashes in breast cancer patients than clonidine	NR
Loprinzi [33]	Venlafaxine in management of hot flashes in survivors of breast cancer	1.Venlafaxine 37.5 mg/day *n* = 56 2.Venlafaxine 75 mg/day *n* = 55 3.Venlafaxine 150 mg/day	Placebo *n* = 56	1. V(37.5 mg) *n* = 7 2.V(75 mg) *n* = 12 3. V(150 mg) *n* = 5	6	Yes/Yes	A-clear	Venlafaxine, 3 doses, 37.5 mg, 75 mg and 150 mg vs placebo. Parallel group design, randomized, double blind	4 weeks	Decrease in hot flash frequency was significant in all three venlafaxine doses compared to placebo. Four different adverse effects were significantly higher in 75 mg and 150 mg groups vs placebo	Public Health Service Grants. USA
Maclaughlan [40]	Hypnotherapy versus gabapentin for the treatment of hot flashes in breast cancer survivors	Hypnoth. * n* = 13	Gabapentin* n* = 14	4	6	Yes/Yes	B- unclear (no blinding, small sample size)	Gabapentin 900 mg/day vs 3 x 1 hour hypnotic inductions, 1 week apart, instruction in self-hypnosis and home CD use. Parallel group design, randomized, non- blinded	8 weeks	Hypnotherapy and gabapentin demonstrate efficacy in improving hot flashes, there were no significant differences between the 2 arms	Not funded
Mao [41]	El.acupuncture versus gabapentin for hot flashes in breast cancer survivors	El.acup.* n* = 62 (real * n* = 30, sham* n* = 32)	Gabapentin 900 mg/day* n* = 58 (real * n* = 28, placebo* n* = 30)	El.acup* n* = 10 (real * n* = 6, sham* n* = 4)	Gaba* n* = 5 (real * n* = 1, placebo* n* = 4)	Yes/Yes	A-clear	Electroacupuncture (real and sham) versus gabapentin (real and placebo). Parallel group design, 4 arms, randomized, double-blind	8 weeks, obs. at week 24	Acupuncture produced larger placebo and smaller nocebo effects than the pills El.acup reduced HF by 47.8%, gabapentin by 39.4%, sham acup by 45% and placbo pills by 22.3%	1. Pfizer 2. Genetech 3. Incyte 4. Millenium Pharmaceut 5. Bayer 6. Veridex 7. Calithera Biosciences 8. Glaxo.S.K. 9. Wyeth
Pandya [37]	Clonidine for tamoxifen-induced hot flashes in breast cancer patients	Clonidine* n* = 99	Placebo* n* = 99	26	23	No/Yes	A-clear	Clonidine 0.1 mg before bed vs placebo. Parallel group design, randomized, double-blind	8 weeks + 4 week obs	Clonidine significantly reduced frequency and severity of hot flashes	National Cancer Institute, Maryland, USA
Pandya [34]	Gabapentin for hot flashes in women with breast cancer. Dose related efficacy and adverse effect profile was assessed	Gabapentin (300 mg)* n* = 139 Gabapentin (900 mg)* n* =144	Placebo * n* = 137	Gaba 300 mg* n* = 25 Gaba 900 mg* n* = 24	24	Yes/Yes	A-clear	Gabapentin 300 mg/day vs 900 mg/day vs placebo. Parallel group design, randomized, double-blind	8 weeks	Gabapentin is effective in the control of hot flashes at 900 mg/day, but not at 300 mg, measured at 8 weeks	US National Cancer Institute
Walker [20]	Acupuncture versus venlafaxine for vasomotor symptoms in patients with hormone receptor positive breast cancer	Acupuncture* n* = 25	Venlafaxine* n* = 25	1	5	No/Yes	B-unclear (not possible to blind providers and participants, possibly affecting bias)	Acupuncture (16 treatments) vs venlafaxine (37.5 mg/day for 1 week, then 75 mg for 11 weeks) Parallel group design, randomized, not blinded	12 weeks, observed for 1 year	Acupuncture and venlafaxine significantly and equally reduced hot flash symptoms. Eighteen incidents of adverse effects were seen in the venlafaxine group, there were none in the acupuncture group	Susan Komen Foundation

The column “Participants” refers to the number of participants randomized to either treatment or control group. “Dropout” refers to participants who left the study in either the treatment or the control group, respectively

### Meta-analysis

Study populations were divided into groups experiencing adverse effects versus those with no adverse effects in both treatment and control groups. Homogenous study designs including participants, interventions, control groups and outcome measures were combined and a meta-analysis performed; *P* < 0.10 defined significant heterogeneity. Odds ratios and 95 % confidence intervals were calculated from the number of patients experiencing adverse effects in each group based on the total number of patients randomized to either treatment or control group. Studies with no adverse effects either in one or both groups were given an added continuity correction of 0.5 in order to estimate a valid approximation of odds ratio [30]. Data regarding the adverse effect in a trial carried out by Boekhout, which compared venlafaxine and clonidine to placebo, were found to be identical for both the venlafaxine and clonidine groups [31]. These data were included only once in the meta-analysis to avoid overrepresentation of adverse effects in the intervention group. Three studies comparing different drug dosages to placebo were divided according to high and low dosage in the meta-analysis [32–34]. Based on the total number of participants randomized to the treatment or control group, odds ratios and 95 % confidence intervals were calculated from the number of patients experiencing adverse effects in each group. To perform a meta-analysis, data were entered directly from the datasheets into Review Manager 5 computer program [35].

## Results

### Outcome of the literature searches

**Fig. 1:**
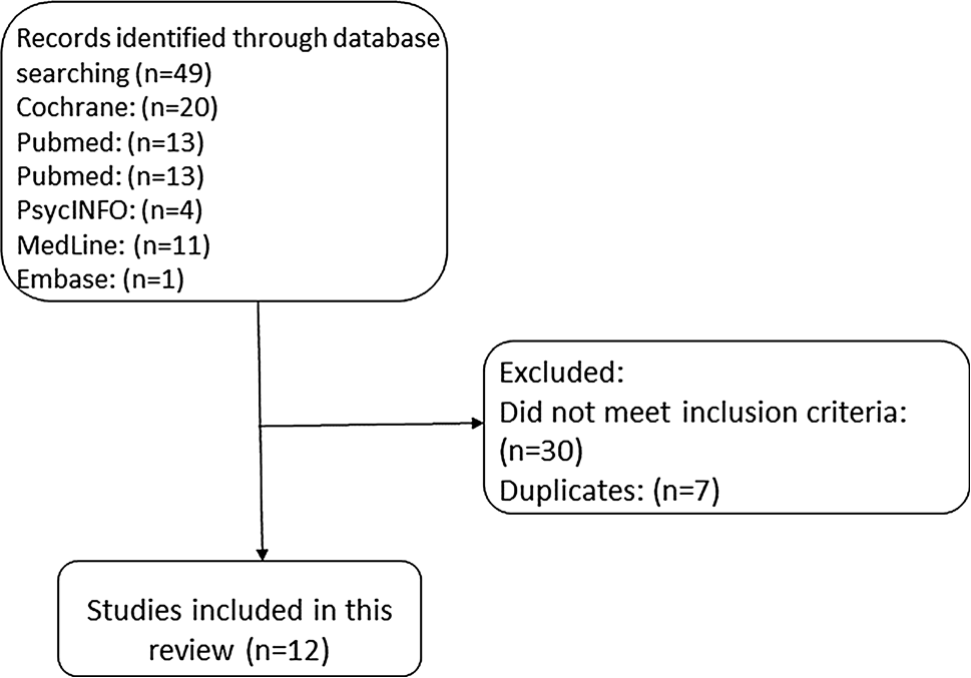
Flow chart for included RCTs

A total of 49 articles were identified. They were initially examined on the basis of titles and abstracts; 37 were excluded from further examination due to the following: 30 did not meet inclusion criteria and seven were multiple article registrations in databases. A total of 12 articles were included in this review (Fig. 1).

The control intervention was clonidine in three studies [31, 37, 39] and placebo in five studies. These five studies compared venlafaxine, or clonidine, or gabapentin to placebo [32–34, 37, 38]. Two of these studies examined venlafaxine at two [32] and three [33] different doses and one study examined gabapentin at two different doses [34]. Three studies compared gabapentin to other therapies: vitamin E [39], hypnotherapy [40] and electro-acupuncture [41], and one compared venlafaxine to acupuncture [20].

Methodological assessment as described in the Cochrane handbook was used to rate the included trials: All were classified as high quality (A), apart from three RCTs in which risk of bias was increased by providers and participants not being blinded [20, 39] and one where no blinding was used and the sample size was small [40].

Five studies included more than one active treatment arm [31–34, 41]. Four studies had a cross-over design [32, 36, 38, 42]. Number of participants ranged from a minimum of 27 to a maximum of 420. The duration of the studies ranged from 4 to 24 weeks.

Types of adverse effects were reported in all the included studies. Number of patients suffering from adverse effects and number of adverse effects were reported in all but two studies [32, 38] where specific adverse effects were compared to placebo and reported as *p* values. We tried to contact the authors of these two studies in order to gain access to more comparable data. We were not able to get in touch with Goldberg; Carpenter kindly provided more data, but the actual numbers concerning adverse effects were not available. These two studies were consequently excluded from the meta-analysis. One study presented data on adverse effects only if these were the reason for dropping out, possibly causing an underestimation of the number of adverse effects [34]. A total of 1467 participants experienced 772 adverse effects. Of these, 81 % (*n* = 627) were in the treatment group and 19 % (*n* = 145) were in the control group. Adverse effects included appetite disorder, nausea, dry mouth, fatigue, dizziness, headache, difficulty sleeping, anxiety, memory problems, sweating, constipation, double vision and increased blood pressure.

Sixty-seven percent of the adverse effects were graded as CTCAE I (*n* = 515) and 33 % were graded as CTCAE II (*n* = 257) (Table 2). Adverse effects causing participants to dropout were classified as CTCAE grade II.

**Table 2:** Adverse effects reported in the randomized controlled trials

Study ID	Number of participants		Total no. of AE (no. of participants with AE)		Type of AE
	Treatment	Control	Treatment	Control	
Biglia [39]	Gabap.* n* = 60	Vit E 55	17 (60)	0 (55)	Somnolence, dizziness, dry mouth, nervousness, weight gain
Boekhout [31]	Venl 41. Clon 41.	Placebo *n* = 20	Venl 206 (41) Clon163 (41)	82 (20)	Reduced appetite, nausea, sleepiness, dizziness, fatigue, dry mouth, sweating, constipation
Buijs [36]	Venlafaxine *n* = 30	Clonidine *n* = 30	27 (30)	5 (30)	Headache, dizziness, dry mouth, mood disorder
Carpenter [32] (high dose)*	Venlafaxine *n* = 9	Placebo *n* = 9	Dry mouth p = 0.002 vs placebo **	NR	Dry mouth
Carpenter [32] (low dose)*	Venlafaxine *n* = 26	Placebo *n* = 26	Constipation* p* = 0.001 headaches* p* = 0.007 dry mouth* p* = 0.001 vs placebo **	NR	Constipation, headache, dry mouth
Goldberg [38]	Clonidine *n* = 55	Placebo *n* = 55	dry mouth (*p* < .001) constipation (.02) itchiness (.01)	NR	Drowsiness, dry mouth, constipation, itching
Loibl [42]	Venlafaxine *n* = 33	Clonidine *n* = 31	38 (27)	14 (27)	Loss of appetite, sleeplessness, nausea, drowsiness, tiredness, sweating, constipation, restless sleep, nervousness, moodiness, dry mouth
Loprinzi [33] (High dose)*	Venlafaxine 150 mg *n* = 54	Placebo *n* = 56	43(54)	2 (56)	Decreased appetite, nausea, dry mouth, constipation
Loprinzi [33] (Low dose) *	Venlafaxine 37.5 mg *n* = 56.	Placebo *n* = 56	14 (56)	2 (56)	Decreased appetite, nausea, dry mouth, constipation
Loprinzi [33](Medium dose)*	Venlafaxine 75 mg *n* = 55.	Placebo *n* = 56	18 (55)	2 (56)	Decreased appetite, nausea, dry mouth, constipation
Maclaughlan [40]	Gabapentine. *n* = 14	Hypnotherapy *n* = 13	3 (24)	0 (13)	Fatigue, vertigo
Mao [41]	Gabapentin 900 mg/day = 28	El.acupuncture = 30	Gabapentin 13 (28)	5 (30)	Bruising, constipation, dizziness, dry mouth, fatigue, headache, increased pain, drowsiness
Pandya [37]	Clonidine *n* = 99	Placebo 99	41 (99)	21 (99)	Difficulty sleeping
Pandya [34] (high dose) *	Gabapentine 900 mg *n* = 116	Placebo *n* = 113	10 (120)	6 (113)	Appetite, distress, drowsiness, fatigue, nausea, pain, memory, shortness of breath, sleep, vomiting
Pandya [34] (low-dose study)*	Gabapentine 300 mg *n* = 114	Placebo *n* = 119	6 (114)	6 (113)	Appetite, distress, drowsiness, fatigue, nausea, pain, memory, shortness of breath, sleep, vomiting
Walker [20]	Venlafaxine* n*= 25	Acupuncture* n*=25	28 (25)	0 (25)	Nausea, dry mouth, headache, difficulty sleeping, double vision, increased blood pressure, constipation, anxiety, lightheaded, jittery
SUM			627 (774)		

Study ID	Grade 1-5 (CTCAE)				
	Treatment				
	G1	G2	G3	G4	G5
Biglia [39]	5	12			
Boekhout [31]	V 104. Cl 93	V. 102, Cl 70			
Buijs [36]	16	11			
Carpenter [32] (high dose)*					
Carpenter [32] (low dose)*					
Goldberg [38]					
Loibl [42]	38				
Loprinzi [33] (High dose)*	40	3			
Loprinzi [33] (Low dose) *	13	1			
Loprinzi [33](Medium dose)*	17	1			
Maclaughlan [40]	3				
Mao [41]	13				
Pandya [37]	41				
Pandya [34] (high dose) *		10			
Pandya [34] (low-dose study)*	6				
Walker [20]	28				
SUM	417	210	0	0	0

Study ID	Grade 1-5 (CTCAE)				
	Control				
	G1	G2	G3	G4	G5
Biglia [39]	0				
Boekhout [31]	40	42			
Buijs [36]		5			
Carpenter [32] (high dose)*					
Carpenter [32] (low dose)*					
Goldberg [38]					
Loibl [42]	14				
Loprinzi [33] (High dose)*	2				
Loprinzi [33] (Low dose) *	2				
Loprinzi [33](Medium dose)*	2				
Maclaughlan [40]					
Mao [41]	5				
Pandya [37]	21				
Pandya [34] (high dose) *	6				
Pandya [34] (low-dose study)*	6				
Walker [20]	0				
SUM	98	47	0	0	0

Whether dropping-out in the included studies was due to adverse effects was reported in all but four studies [33, 34, 38, 41]. In the three studies comparing venlafaxine and clonidine, the number of dropouts due to adverse effects were fourteen and five [36], six and four [42] and six and two [31], respectively, totalling 26 in the venlafaxine groups versus 11 in the clonidine groups. Gabapentin was compared to placebo [34], hypnotherapy [40] and vitamin E [39], where sixteen, three and seventeen women, respectively, dropped out of the gabapentin groups due to adverse effects; there were no dropouts in the second arms. Venlafaxine was compared to placebo [32], where the number of dropouts were 3 versus 1, and acupuncture [20], where the only dropouts due to adverse effects were 3 women in the venlafaxine group.

### Meta-analyses

Adverse effects’ data from 10 RCTs were included in the meta-analysis with a total of 1,428 subjects.

#### Non-hormonal medication versus overall control

An overall comparison was made between non-hormonal medication and control. Ten trials had 13 different outcomes due to low and high drug doses in the same trials. A significant difference was found between non-hormonal medication and control, with OR of 1.67, 95 % CI of 1.31–2.13 and *I* ^2^ of 85 % (*P* < 0.0001).

Different sub-group meta-analyses according to the categories of controls were performed, and are presented below.

#### Non-hormonal medication versus placebo

A comparison was made between non-hormonal medication and placebo. Two trials (259 participants) made this comparison, and a statistically significant difference was found between non-hormonal medication and placebo, with OR of 1.51, 95 % CI of 1.16–1.98 and *I* ^2^ of 0 % (*P* = 0.002).

#### High-dose non-hormonal medication versus placebo

There was no statistically significant difference between high-dose non-hormonal medication and placebo in a meta-analysis of two trials (*n* = 450) for three different combined outcomes, with OR of 2.96, 95 % CI of 0.97–9.05 *I* ^2^ and 93 % (*P* = 0.06).

#### Low-dose non-hormonal medication versus placebo

A comparison was made between low-dose non-hormonal medication and placebo. Two trials (345 participants) made this comparison, and no statistically significant difference was found between the groups (OR 1.53, 95 % CI 0.62–3.77, *I* ^2^ = 87 %, *P* = 0.36).

#### Non-hormonal medication versus non-hormonal medication

There was a significant difference between non-hormonal medication (venlafaxine) and non-hormonal medication (clonidine) in a meta-analysis of two trials, with OR of 1.44, 95 % CI of 1.00–2.08 and *I* ^2^ of 45 % (*P* = 0.05).

#### Non-hormonal medication versus acupuncture

A comparison was made between non-hormonal medication and acupuncture. Two trials (108 participants) made this comparison; a significant difference was found between the groups in favour of acupuncture, with OR of 1.75, 95 % CI of 01.09–2.75 and *I* ^2^ of 0 % (*P* = 0.02).

#### Non-hormonal medication versus other therapy

There was no statistically significant difference between non-hormonal medication and other therapies in a meta-analysis of two trials, with OR of 1.34, 95 % CI of 0.74–2.45 and *I* ^2^ of 34 % (*P* = 0.33).

## Discussion

This meta-analysis demonstrated that the odds for experiencing adverse effects was significantly higher in patients randomized to non-hormonal medication than for patients randomized to controls, such as placebo and acupuncture. High-dose non-hormonal medication (venlafaxine and gabapentin) provoked an increased number of adverse effects compared to low-dose medication. This may suggest that low-dose non-hormonal medication is a good alternative for breast cancer survivors with hot flashes, providing sufficient reduction in frequency and intensity of hot flashes. Rada et al. [8] in their systematic review report that non-hormonal therapies have a mild to moderate effect in reducing frequency and intensity of hot flashes in women with a history of breast cancer. This result was based on nine different studies evaluating the effect of SSRIs (*n* = 6), clonidine (*n* = 2) and gabapentin (*n* = 1).

Acupuncture has few adverse effects compared to non-hormonal medication and should be considered as a potential treatment alternative if efficacy can be confirmed in future studies. Four systematic reviews evaluating acupuncture for hot flashes in breast cancer survivors included six [44], seven [43], eight [21] and twelve [45] RCT’s respectively. Overall, authors concluded that acupuncture effectively reduced hot flashes, but was not statistically significant compared to sham; and that there is currently insufficient evidence to either support or refute acupuncture for this patient category.

Twelve trials were identified for this systematic review, and ten of these were included in the meta-analysis. We pooled results in an attempt to give an overall comparison of non-hormonal medication versus control; six different sub-group analyses were done. However, only two trials made up each group, thereby only demonstrating tendencies.

### Study strengths and limitations

As far as we know, this is the first systematic review and meta-analysis to examine adverse effects of non-hormonal medications for hot flashes in breast cancer survivors, as the primary outcome measure. The included studies were of high methodological quality and with reduced risk of bias, thereby providing reliable results. Heterogeneity is always an important consideration when compared to RCTs, and the forest plot showed strong study similarities.

Two-thirds of adverse effects reported in this review were classified as grade I and a third as grade II. The CTCAE grading of adverse effects was solely based on information provided by the articles included in this review, and should be considered as an approximation of adverse effect severity. Inconsistent use of safety terminology made it difficult to categorize and evaluate the data; the CTCAE grading system was not consistently used.

Three different non-hormonal medications were assessed and compared to different control groups. This was a limiting factor in the meta-analysis. To reduce the risk of inflating the size of the pooled treatment effect, zero-cell counts were included [46]. A continuity correction of 0.5 was used for studies with zero-cell counts, in order to provide a conservative approximation of adverse event risk [47].

**Fig. 2:**
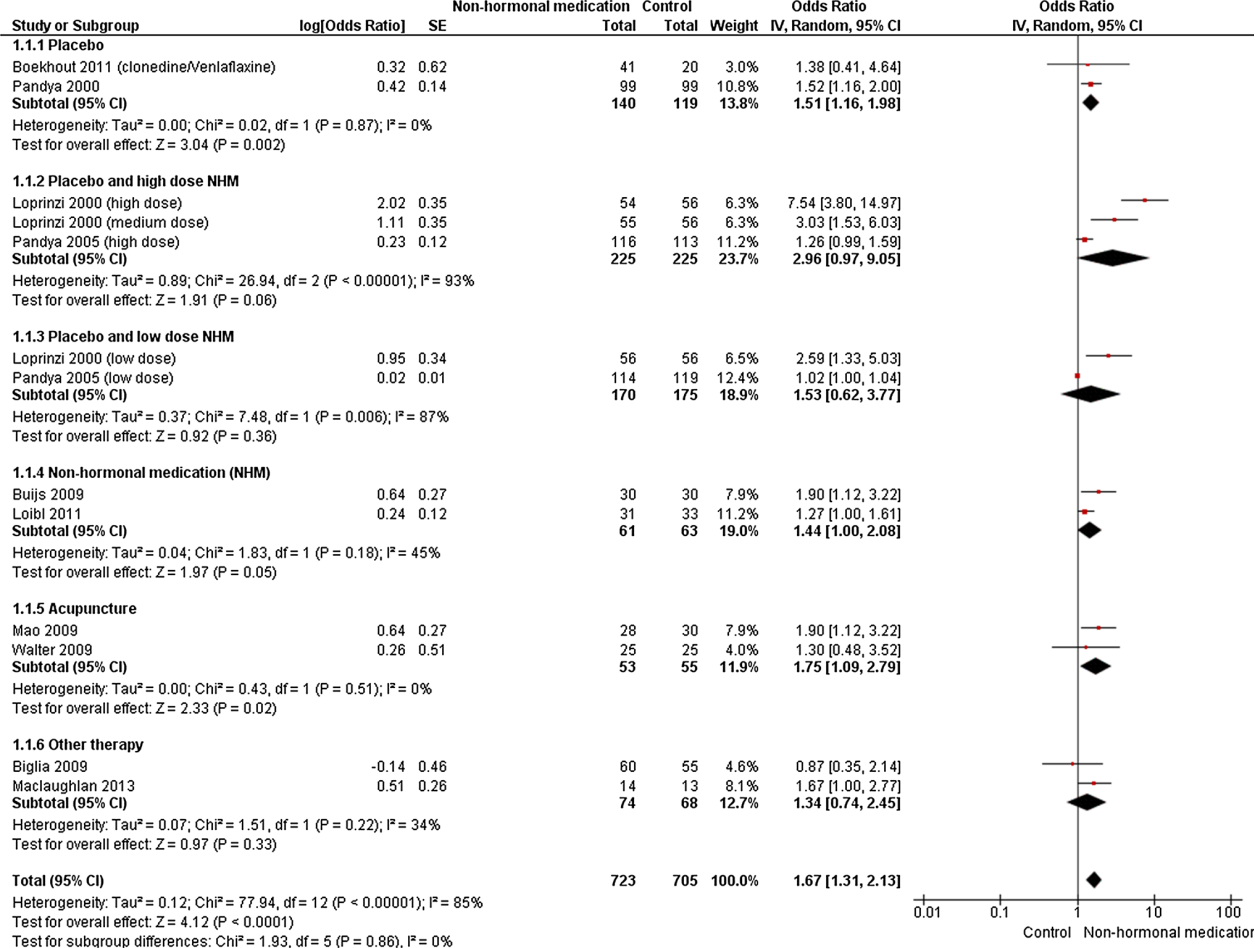
Forest Plot

Six studies included in the meta-analysis had active controls, including other non-hormonal medicines, acupuncture and other therapies, possibly inflating adverse-effect frequency outcomes; however, the forest plot (Fig. 2) does not indicate such influence when studies with active controls are compared to those with passive controls.

Other elements of conceivable bias include possible under-reporting of adverse effects by participants motivated to experience treatment effect, simply due to being included in a clinical trial. Publication bias is also a consideration; clinical trials demonstrating a statistically significant treatment effect compared to control are more likely to be published [48].

Search strategy for this review included five search engines, and more RCTs may have been identified if more search engines had been added. However, we also identified a systematic review focusing on active interventions for hot flash symptoms in breast cancer patients [8] and 4 reviews focusing on a combination of post-menopausal women and breast cancer survivors [9, 26–28]. Examination of the full texts and reference lists of these reviews did not provide any additional RCTs for this meta-analysis.

### Other studies

To our knowledge, only one systematic review evaluating non-hormonal therapies for hot flashes in women with a history of breast cancer has been published, and the focus was on treatment efficacy [8]. We could not find any systematic reviews that examined adverse effects due to non-hormonal drugs as a primary outcome in this patient category. Rada and colleagues reported evidence supporting the use of clonidine, gabapentin and SSRIs/SNRIs for hot flash symptoms in breast cancer survivors. The authors commented that adverse effects were inconsistently reported. 16 studies were included, of which 10 were pharmacological studies and 6 non-pharmacological studies. They confirmed our findings that adverse effects increase when higher doses of gabapentin and venlafaxine were used. They also suggested that adverse effects may outweigh benefit in clonidine.

Another systematic review of 13 randomized trials comparing active interventions for hot flash problems in women with and without breast cancer [26] did not agree with our findings of dose-related increased frequency of adverse effects. The authors reported that high doses of venlafaxine (75 mg/day) and gabapentin (900 mg/day) appeared to improve hot flash symptoms to a greater extent compared to lower doses, without incurring more adverse effects. Since the population did not only include breast cancer patients, the results make comparison with our study difficult.

### Implications

Despite these limitations, the sub-group analyses provided information relevant for clinical practice, including the relationship between drug dosage and adverse effects, drug comparisons in relation to adverse effects and the possible role of acupuncture as a treatment for hot flashes if efficacy can be confirmed. Further research is indicated to investigate these findings with focus on efficacy versus adverse effects; also the effect of combined therapies should be considered with a view to increasing the compliancy of oestrogen-antagonist medication.

## Conclusion

The odds for experiencing adverse effects was statistically significantly higher in patients randomized to high-dose non-hormonal medication than for patients randomized to controls, such as placebo, low-dose medication and acupuncture. Consequently, these therapies should be considered as a potential treatment alternative if efficacy for hot flushes can be confirmed.
